# Efficacy and safety of dual antiplatelet therapy after percutaneous coronary drug-eluting stenting: A network meta-analysis

**DOI:** 10.1097/MD.0000000000031158

**Published:** 2022-10-21

**Authors:** Lin Luo, Shenglin Wang, Kai Tang, Xu Yang, Jianli Wu, Dan Wang, Liqiong Xu, Tao Feng, Dejin Li, Jiuju Ran, Debo Li, Li Zhang, Dan Zhao

**Affiliations:** a Department of Cardiovascular, The First People’s Hospital of Shuangliu District, Chengdu, China; b Department of Neurology, The First Affiliated Hospital of Chongqing Medical University, Chongqing, China; c Department of Ophthalmolgy, Sichuan Academy of Medical Sciences, Sichuan Provincial People’s Hospital, Chengdu, China; d Department of Neurology, The First People’s Hospital of Shuangliu District, Chengdu, China.

**Keywords:** drug-eluting stents, dual antiplatelet antiplatelets, efficacy, network meta-analysis, percutaneous coronary intervention, safety

## Abstract

To evaluate the efficacy and safety of dual antiplatelet regimens after coronary drug-eluting stenting by network meta-analysis (NMA).

**Methods:**

PubMed, The Cochrane Library, Embase, and Web of Science databases were electronically searched to collect randomized controlled trials (RCTs) of the comparison of different dual antiplatelet regimens after coronary drug-eluting stenting from inception to September 1st, 2021. Two reviewers independently screened literature, extracted data, and assessed the risk bias of included studies. Stata 16.0 software was used for NMA.

**Results:**

A total of 27 RCTs involving 79,880 patients were included. The results of NMA: in terms of myocardial infarction (MI), other 3 interventions were higher than the long-term dual antiplatelet therapy (L-DAPT) (the standard dual antiplatelet therapy [Std-DAPT] [odds ratio [OR] = 1.82, 95%confidence interval [CI]: 1.49-2.21), the aspirin monotherapy after short-term dual antiplatelet therapy (S-DAPT + As) (OR = 2.06, 95%CI: 1.57-2.70), the P2Y12 inhibitor monotherapy after short-term dual antiplatelet therapy (S-DAPT + P2Y12) (OR = 1.71, 95%CI: 1.29-2.28)]. In terms of stent thrombosis, other 3 interventions were higher than L-DAPT [Std-DAPT (OR = 2.18, 95%CI: 1.45-3.28), S-DAPT + As (OR = 2.32, 95%CI: 1.52-3.54), S-DAPT + P2Y12 (OR = 2.31, 95%CI: 1.22-4.36)]. There was no statistically significant difference among the 4 interventions in prevention of stroke and all-cause mortality (*P* > .05). In terms of cardiovascular and cerebrovascular adverse events, other 3 interventions were higher than L-DAPT (Std-DAPT [OR = 1.28, 95%CI: 1.12-1.45], S-DAPT + As [OR = 1.27, 95%CI: 1.09-1.48], S-DAPT + P2Y12 [OR = 1.24, 95%CI: 1.01-1.52]). In terms of safety, bleeding rate of other 3 interventions were lower than L-DAPT (Std-DAPT [OR = 0.67, 95%CI: 0.52-0.85], S-DAPT + As [OR = 0.51, 95%CI: 0.39-0.66], S-DAPT + P2Y12 [OR = 0.36, 95%CI: 0.26-0.49]). Two interventions were lower than L-DAPT (S-DAPT + As [OR = 0.77, 95%CI: 0.65-0.90], S-DAPT + P2Y12 [OR = 0.54, 95%CI: 0.44-0.66]). S-DAPT + As was higher than L-DAPT (OR = 1.42, 95%CI: 1.10-1.83).

**Conclusions:**

S-DAPT + P2Y12 has the lowest bleeding risk, while L-DAPT has the highest bleeding risk. In the outcome of MI, stent thrombosis, and cardiovascular and cerebrovascular adverse events, L-DAPT has the best efficacy. In the outcome of stroke and all-cause mortality, the 4 interventions were equally effective.

## 1. Introduction

Compared with the original bare metal stent, drug-eluting stent (DES) has greatly improved the risk of in-stent restenosis and target lesion revascularization after percutaneous coronary intervention (PCI).^[1–3]^ Concerningly, DES may be associated with the risk of late stent thrombosis,^[4,5]^ although stent thrombosis events have become relatively rare in newer-generation DES, it still threatens the lives of patients today and cannot be ignored.

Dual antiplatelet therapy (DAPT) of aspirin combined with P2Y12 receptor inhibitors is the cornerstone of post-PCI treatment, which is crucial to prevent stent thrombosis and can effectively reduce the occurrence of ischemic events.^[6]^ The 2016 ACC/AHA, 2016 China and 2017 ESC guidelines for PCI^[7–9]^ all recommend that patients with acute coronary syndrome (ACS) receive standard dual antiplatelet therapy (Std-DAPT) for at least 12 months after DES implantation. Patients with stable ischemic heart disease without high bleeding risk may receive aspirin monotherapy after 1-3 months of short-term dual antiplatelet therapy. Patients with ACS without high bleeding risk may receive 6 months of dual antiplatelet therapy (S-DAPT + As).

 However, in patients at higher ischemic risk for ACS, DAPT reduces ischemic events at the expense of increased bleeding risk.^[10,11]^ In recent years, in order to balance the efficacy and safety of DAPT, some researchers recommend discontinuing aspirin after short-term dual antiplatelet and continuing to use P2Y12 inhibitor monotherapy (S-DAPT + P2Y12) to shorten the DAPT time after PCI to reduce bleeding events.^[12]^ Some researchers have suggested that the occurrence of stent thrombosis in late ischemic event may be related to delayed coronary endothelial healing after DES implantation,^[13]^ and premature discontinuation of DAPT treatment has been identified as a risk factor for late stent thrombosis after DES implantation.^[14,15]^ Long-term (more than 12 months) dual antiplatelet therapy (L-DAPT) may be recommended to reduce ischemic events if there is no high risk of bleeding.^[16]^

At present, there is a lack of comparison on the efficacy and safety of different DAPT regimens. This study will use network meta-analysis (NMA) to evaluate different DAPT regimens, to provide the evidence-based basis for clinical workers in the future.

## 2. Methods

### 2.1. Data sources and search strategy

Because the NMA is a secondary analysis study, it does not involve ethical approval. PubMed, The Cochrane Library, Embase, and Web of Science databases were randomized searched by computer to collect controlled trials (RCTs) on the comparison of different dual antiplatelet regimens after coronary drug-eluting stenting from the establishment of the database to September 1st, 2021. At the same time, reference literatures of published studies were traced back, and paper versions of relevant conferences were manually read to supplement (Details of our search strategy are provided in the Supplementary Appendix, http://links.lww.com/MD/H627).

### 2.2. Study selection

All studies must be RCTs. Patients with DES implantation who satisfied the clinical diagnostic criteria for coronary heart disease were over 18 years old. The follow-up period was at least 12 months. The experimental group and control groups were treated with different DAPT, which was aspirin combined with P2Y12 receptor inhibitors (such as clopidogrel, ticlopidine, ticagrelor, and prasugrel).

### 2.3. Data extraction and quality assessment

Two researchers independently screened studies, extracted data, and cross-checked them. Disputes, if any, shall be resolved through discussion or consultation with a third party. When screening studies, read the title first, then the abstract and entire text to determine whether to include it. To get information, contact the original study author by email or phone if necessary. The RCT bias risk assessment tool recommended by Cochrane Manual 5.1.0 was used to assess the risk of bias.^[17]^

The following data were recorded: publication characteristics, countries or regions of the study, patient characteristics, classification of coronary heart disease, sample size, interventions, duration of follow-up, blinding, intention-to-treat analysis, efficacy and safety outcomes. The efficacy outcomes included myocardial infarction (MI), stent thrombosis, stroke, all-cause mortality, cardiovascular and cerebrovascular adverse events. The safety outcome included bleeding.

### 2.4. Data analysis

A random-effect model was constructed based on frequency theory, and Stata16.0 software was used for direct and NMA. *χ*^2^ test and *I*^2^ value were used to determine heterogeneity. If there was significant heterogeneity between studies, the source of heterogeneity was analyzed first. The outcome indicators were odds ratio (OR) for dichotomous variables and mean difference for continuous variables, with a 95% confidence interval (CI) as the test level. If the number of included studies was greater than 10, a funnel plot was made to evaluate whether the intervention had a small sample effect and publication bias. The network plot represents the sample size and relationship of the interventions. When there was a closed loop, the inconsistency evaluation method was used to test the inconsistency. If the difference was not statistically significant (*P* > .05), and the consistency model was used for analysis. At the same time, the node-splitting method was used to test the local inconsistency. By surface under the cumulative ranking (SUCRA), the advantages and disadvantages of therapies were quantitatively compared. The larger SUCRA was, the more likely the treatment was to become the best treatment. Then, the efficacy of different therapies could be compared comprehensively.

## 3. Results

### 3.1. Study selection

**Figure 1.**
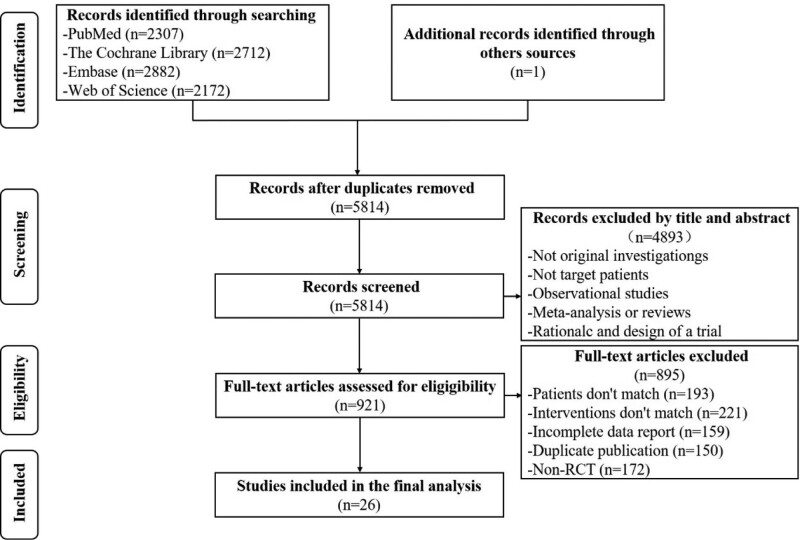
Study selection flow diagram.

A total of 10,074 related studies were obtained in the preliminary review. Twenty-six studies were eventually included,^[18–43]^ including 1 conference paper containing 2 RCTs.^[29]^ The 27 RCTs included a total of 79,880 patients (Study selection flow diagram in Fig. 1).

### 3.2. Quality evaluation and baseline characteristics

**Figure 2.**
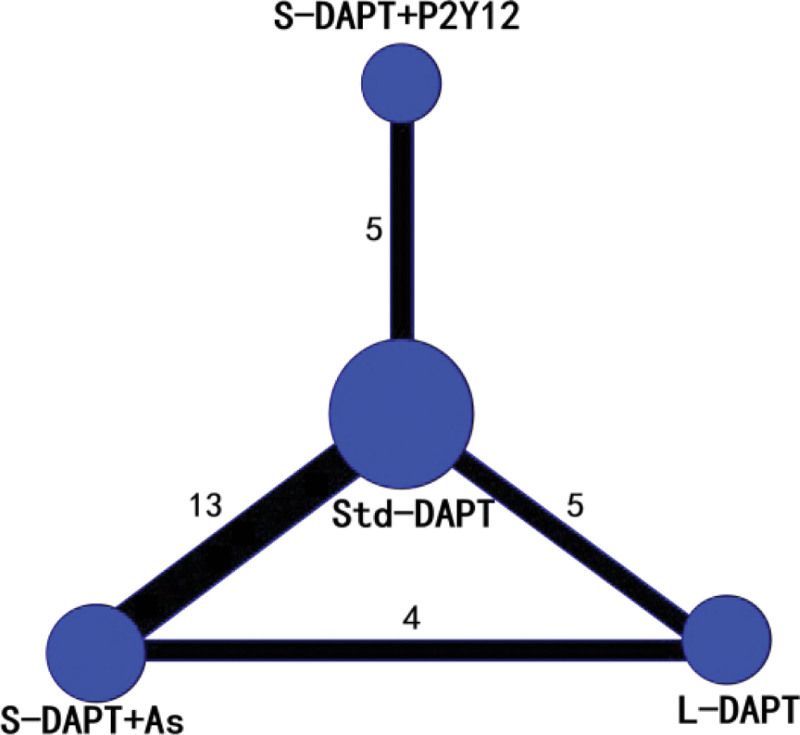
The network plot.

Random sequence generation, double-blind, allocation concealment, and complete outcome data were used in the majority of the studies (Quality evaluation in Table 1). A total of 4 interventions were included (Baseline characteristics in Table 2 and the network plot presented in Fig. 2). There were 13 RCTs comparing S-DAPT + As with Std-DAPT.^[18–29]^ There were 5 RCTs comparing S-DAPT + P2Y12 with Std-DAPT.^[30–34]^ There were 5 RCTs comparing L-DAPT with Std-DAPT.^[35–39]^ There were 4 RCTs comparing S-DAPT + As with L-DAPT.^[40–43]^

**Table 1:** The result of the quality evaluation.

Study ID	Random method	Blinding	Allocation plan hidden	Integrity of the result data	Selective reporting	Other sources of bias
ISAR-SAFE 2015^[18]^	Computer	Double-blind	Sealed envelope	Basically complete*, non-ITT	No	Not sure
OPTIMA-C 2018^[19]^	Computer	Open-label	Interactive-response computer system	Basically complete*, non-ITT	No	Not sure
RESET 2012^[20]^	Computer	Open-label	Interactive-response computer system	Basically complete*, ITT	No	Not sure
DAPT-STEMI 2018^[21]^	Not sure	Open-label	Not sure	Basically complete*, ITT	No	Not sure
IVUS-XPL 2016^[22]^	Computer	Not sure	Interactive-response computer system	Basically complete*, ITT	No	Not sure
I-LOVE-IT 2 2016^[23]^	Not sure	Investigator blinding	Not sure	Basically complete*, ITT	No	Not sure
EXCELLENT 2012^[24]^	Computer	Open-label	Interactive-response computer system	Basically complete*, ITT	No	Not sure
OPTIMIZE 2013^[25]^	Not sure	Open-label	Not sure	Basically complete*, non-ITT	No	Not sure
REDUCE 2019^[26]^	Computer	Open-label	Interactive-response computer system	Basically complete*, non-ITT	No	Not sure
SECURITY 2014^[27]^	Not sure	Investigator blinding	Not sure	Basically complete*, ITT	No	Not sure
ONE-MONTH DAPT 2021^[28]^	Not sure	Open-label	Center allocation	Basically complete*, ITT	No	Not sure
XIENCE 90 2020^[29]^	Not sure	Not sure	Not sure	Not sure	Not sure	Conference paper
XIENCE 28 2020^[29]^	Not sure	Not sure	Not sure	Not sure	Not sure	Conference paper
TWILIGHT 2019^[30]^	Computer	Double-blind	Not sure	Basically complete*, ITT	No	Not sure
STOPDAPT-2 2019^[31]^	Computer	Investigator blinding	Center allocation	Basically complete*, non-ITT	No	Not sure
SMART-CHOICE 2019^[32]^	Computer	Open-label	Interactive-response computer system	Basically complete*, ITT	No	Not sure
GLOBAL LEADER 2019^[33]^	Computer	Open-label	Center allocation	Basically complete*, non-ITT	No	Not sure
TICO 2020^[34]^	Computer	Open-label	Interactive-response computer system	Basically complete*, ITT	No	Not sure
REAL-LATE and ZEST-LATE 2010^[35]^	Computer	Open-label	Center allocation	Not sure	No	Two RCTs merged
DAPT 2014^[36]^	Not sure	Open-label	Center allocation	Basically complete*, ITT	No	Not sure
DES-LATE 2014^[37]^	Computer	Open-label	Not sure	Basically complete*, ITT	No	Not sure
OPTIDUAL 2016^[38]^	Computer	Open-label	Interactive-response computer system	Basically complete*, ITT	No	Not sure
ARCTIC 2014^[39]^	Computer	Open-label	Interactive-response computer system	Basically complete*, non-ITT	No	Not sure
NIPPON 2017^[40]^	Computer	Not sure	Interactive-response computer system	Basically complete*, ITT	No	Not sure
SMART-DATE 2018^[41]^	Computer	Open-label	Center allocation	Basically complete*, ITT	No	Not sure
ITALIC 2015^[42]^	Computer	Open-label	Interactive-response computer system	Basically complete*, non-ITT	No	Not sure
PRODIGY 2014^[43]^	Not sure	Not sure	Not sure	Basically complete*, non-ITT	No	Not sure

* The study was lost to follow-up, but the number of lost to follow-up in each group was balanced, or the proportion of lost to follow-up was very low, which had little impact on the completeness of the result data.

ITT = intentional analysis.

**Table 2:** The result of the baseline characteristics.

Study ID	Country/region	N patients (T/C)	Average age (yrs)	Hypertension (%)	Diabetes (%)	Interventions		Treatment time (T vs C) (mo)	Follow-up time (mo)	Outcome
						T	C			
ISAR-SAFE 2015^[18]^	Global	1997/2003	67.2 ± 6.5	91	24	AS and PL	CL and AS	6 vs 12	15	abcdef
OPTIMA-C 2018^[19]^	South Korea	683/684	63.0 ± 11.0	63	29	AS	CL and AS	6 vs 12	12	adef
RESET 2012^[20]^	South Korea	1059/1058	62.4 ± 9.8	62	30	AS	CL and AS	3 vs 12	12	abdef
DAPT-STEMI 2018^[21]^	Europe	433/437	60.0 ± 10.7	45	14	AS	CL or PR or TI and AS	6 vs 12	24	abcdef
IVUS-XPL 2016^[22]^	South Korea	699/701	63.5 ± 9.0	64	37	AS	CL and AS	6 vs 12	12	bcdef
I-LOVE-IT 2 2016^[23]^	China	909/920	60.2 ± 10.0	63	23	AS	CL and AS	6 vs 12	12	abcdef
EXCELLENT 2012^[24]^	South Korea	722/721	62.7 ± 10.0	73	38	AS	CL and AS	6 vs 12	12	abdef
OPTIMIZE 2013^[25]^	Brazil	1563/1556	61.6 ± 10.6	87	35	AS	CL and AS	3 vs 12	12	abcdef
REDUCE 2019^[26]^	Europe and Asia	733/727	60.5 ± 8.0	51	21	AS	CL or PR or TI and AS	3 vs 12	12	abcdef
SECURITY 2014^[27]^	Europe	682/717	65.2 ± 10.1	73	31	AS	CL or PR or TI and AS	6 vs 12	12	abcdef
ONE-MONTH DAPT 2021^[28]^	South Korea	1507/1513	67 ± 10	66.5	37.5	AS	CL and AS	1 vs 6-12	12	abcdef
XIENCE 90 2020^[29]^	America	1693/1280	75.3 ± 9.3	90	39	AS	CL or PR or TI and AS	3 vs 12	12	bef
XIENCE 28 2020^[29]^	Global	1392/1411	76.0 ± 8.4	85	37	AS	CL or PR or TI and AS	1 vs 12	12	bef
TWILIGHT 2019^[30]^	Europe	3555/3564	65.2 ± 10.3	72	37	TI and PL	TI and AS	3 vs 12	18	abcdef
STOPDAPT-2 2019^[31]^	Japan	1500/1509	68.6 ± 10.9	74	39	CL	CL and AS	1 vs 12	12	abcdef
SMART-CHOICE 2019^[32]^	South Korea	1495/1498	64.5 ± 10.7	62	38	CL or PR or TI	CL or PR or TI and AS	3 vs 12	12	abcdef
GLOBAL LEADER 2019^[33]^	Europe	3750/3737	64	68	22	TI	TI and AS	1 vs 12	12	abcdef
TICO 2020^[34]^	South Korea	1527/1529	61.0 ± 11.0	51	27	TI	TI and AS	3 vs 12	12	abcdef
REAL-LATE and ZEST-LATE 2010^[35]^	South Korea	1357/1344	62.0 ± 9.8	57	26	CL and AS	AS	36 vs 12	36	abcdef
DAPT 2014^[36]^	America	5020/4941	61.7 ± 10.2	75	31	CL or PR and AS	AS and PL	30 vs 12	33	abcdef
DES-LATE 2014^[37]^	South Korea	2531/2514	62.4 ± 10.1	58	28	CL and AS	AS	36 vs 12	36	abcdef
OPTIDUAL 2016^[38]^	France	695/690	64.1 ± 10.6	59	31	CL and AS	AS	48 vs 12	48	abcdef
ARCTIC 2014^[39]^	France	635/624	64.0 ± 7.0	61	34	CL or PR and AS	AS	18-30 vs 12	30	acdef
NIPPON 2017^[40]^	Japan	1654/1653	67.3 ± 9.7	72	38	AS	CL or Tic and AS	6 vs 18	18	abcdef
SMART-DATE 2018^[41]^	South Korea	1357/1355	62.5 ± 10.0	48	28	AS	CL or PR and AS	6 vs 12-18	18	abcdef
ITALIC 2015^[42]^	Europe	912/910	61.6 ± 11.0	65	37	AS	CL or PR or TI and AS	6 vs 24	24	abdef
PRODIGY 2014^[43]^	Italy	114/110	69.0 ± 10.0	72	32	AS	CL and AS	6 vs 24	24	abdef

a: Myocardial infarction; b: Stent thrombosis; c: Stroke; d: All-cause mortality; e: Cardiovascular and cerebrovascular adverse events; f: Bleeding.

AS = aspirin, C = control group, CL = clopidogrel, PL = placebo, PR = prasugrel, T = test group, TI = ticagrelor, TIc = ticlopidine.

### 3.3. Data consistency and inconsistency test

The evaluation of inconsistencies for 6 outcomes performed using loop-specific heterogeneity estimates revealed 1 triangular loop [(S-DAPT + As)-(Std-DAPT)-(L-DAPT)], all with no significant results (all *P* values > 0.182). The node-splitting method did not yield significant results (all *P* values > .182) (The result of direct and NMA in Table 3).

**Table 3:** The result of direct and network meta-analysis.

Outcome	Interventions	Number of studies	Heterogeneity test		Direct meta-analysis OR (95%CI)	Network meta-analysis OR (95%CI)
			*P* value	*I^2^* value		
Myocardial infarction	S-DAPT + As vs *Std*-DAPT	10^[18–21,23–28]^	.88	0%	1.11(0.88,1.40)	1.13 (0.91, 1.41)
	S-DAPT + P2Y12 vs *Std*-DAPT	5^[30–34]^	.62	0%	0.94(0.76,1.16)	0.94 (0.76, 1.16)
	*Std*-DAPT vs L-DAPT	5^[35–39]^	.13	44%	1.79(1.46,2.19)※	1.82 (1.49, 2.21)※
	S-DAPT + As vs L-DAPT	4^[40–43]^	.83	0%	2.40(1.38,4.19)※	2.06 (1.57, 2.70) ※
	S-DAPT + P2Y12 vs L-DAPT	0	-	-	-	1.71 (1.29, 2.28)※
	S-DAPT + As vs S-DAPT + P2Y12	0	-	-	-	1.20 (0.89, 1.63)
Stent thrombosis	S-DAPT + As vs *Std*-DAPT	12^[18,20–29]^	.88	0%	1.22(0.86,1.74)	1.06 (0.76, 1.50)
	S-DAPT + P2Y12 vs *Std*-DAPT	5^[30–34]^	.57	0%	1.06(0.66,1.73)	1.06 (0.62, 1.80)
	*Std*-DAPT vs L-DAPT	4^[35–38]^	.13	47%	2.59(1.77,3.79)※	2.18 (1.45, 3.28)※
	S-DAPT + As vs L-DAPT	4^[40–43]^	.95	0%	1.79(1.02,3.15)※	2.32 (1.52, 3.54)※
	S-DAPT + P2Y12 vs L-DAPT	0	-	-	-	2.31 (1.22, 4.36)※
	S-DAPT + As vs S-DAPT + P2Y12	0	-	-	-	1.00 (0.54, 1.86)
Stroke	S-DAPT + As vs *Std*-DAPT	8^[18,21–23,25–28]^	.76	0%	0.97(0.65,1.45)	0.92 (0.63, 1.33)
	S-DAPT + P2Y12 vs *Std*-DAPT	5^[30–34]^	.10	48%	1.12(0.65,1.92)	1.11 (0.76, 1.63)
	*Std*-DAPT vs L-DAPT	5^[35–39]^	.56	0%	1.03(0.75,1.42)	0.99 (0.73, 1.35)
	S-DAPT + As vs L-DAPT	2^[40,41]^	.02	82%	0.35(0.02,5.43)	0.91 (0.58, 1.43)
	S-DAPT + P2Y12 vs L-DAPT	0	-	-	-	1.11 (0.68, 1.80)
	S-DAPT + As vs S-DAPT + P2Y12	0	-	-	-	0.82 (0.48, 1.41)
All-cause mortality	S-DAPT + As vs *Std*-DAPT	11^[18–28]^	.80	0%	0.84(0.66,1.07)	0.95 (0.75, 1.21)
	S-DAPT + P2Y12 vs *Std*-DAPT	5^[30–34]^	.55	0%	0.84(0.66,1.06)	0.85 (0.65, 1.11)
	*Std*-DAPT vs L-DAPT	5^[35–39]^	.23	29%	0.85(0.64,1.12)	0.96 (0.72, 1.28)
	S-DAPT + As vs L-DAPT	4^[40–43]^	.10	52%	1.46(0.80,2.67)	0.92 (0.68, 1.23)
	S-DAPT + P2Y12 vs L-DAPT	0	-	-	-	0.81 (0.54, 1.22)
	S-DAPT + As vs S-DAPT + P2Y12	0	-	-	-	1.13 (0.78, 1.62)
Cardiovascular and cerebrovascular adverse events	S-DAPT + As vs *Std*-DAPT	13^[18–29]^	.85	0%	0.99(0.89,1.10)	1.00 (0.90, 1.11)
	S-DAPT + P2Y12 vs *Std*-DAPT	5^[30–34]^	.40	0%	0.97(0.83,1.14)	0.97 (0.83, 1.14)
	*Std*-DAPT vs L-DAPT	5^[35–39]^	.30	18%	1.26(1.09,1.44)※	1.28 (1.12, 1.45) ※
	S-DAPT + As vs L-DAPT	4^[40–43]^	.40	0%	1.38(1.03,1.86)※	1.27 (1.09, 1.48)※
	S-DAPT + P2Y12 vs L-DAPT	0	-	-	-	1.24 (1.01, 1.52) ※
	S-DAPT + As vs S-DAPT + P2Y12	0	-	-	-	1.03 (0.85, 1.24)
Bleeding	S-DAPT + As vs *Std*-DAPT	13^[18–29]^	.82	0%	0.73(0.63,0.84)※	0.77 (0.65, 0.90) ※
	S-DAPT + P2Y12 vs *Std*-DAPT	5^[30–34]^	.56	0%	0.54(0.47,0.64)※	0.54 (0.44, 0.66) ※
	*Std*-DAPT vs L-DAPT	5^[35–39]^	.11	47%	0.56(0.46,0.67)※	0.67 (0.52, 0.85)※
	S-DAPT + As vs L-DAPT	4^[40–43]^	.74	0%	0.68(0.48,0.97)※	0.51 (0.39, 0.66)※
	S-DAPT + P2Y12 vs L-DAPT	0	-	-	-	0.36 (0.26, 0.49)※
	S-DAPT + As vs S-DAPT + P2Y12	0	-	-	-	1.42 (1.10, 1.83)※

L-DAPT = Long-term dual antiplatelet therapy, S-DAPT + As = aspirin monotherapy after short-term dual antiplatelet, S-DAPT + P2Y12 = P2Y12 inhibitor monotherapy after short-term dual antiplatelet, *Std*-DAPT = standard dual antiplatelet.

※The difference was statistically significant.

### 3.4. Network meta-analysis

#### 3.4.1. Myocardial infarction.

**Figure 3.**
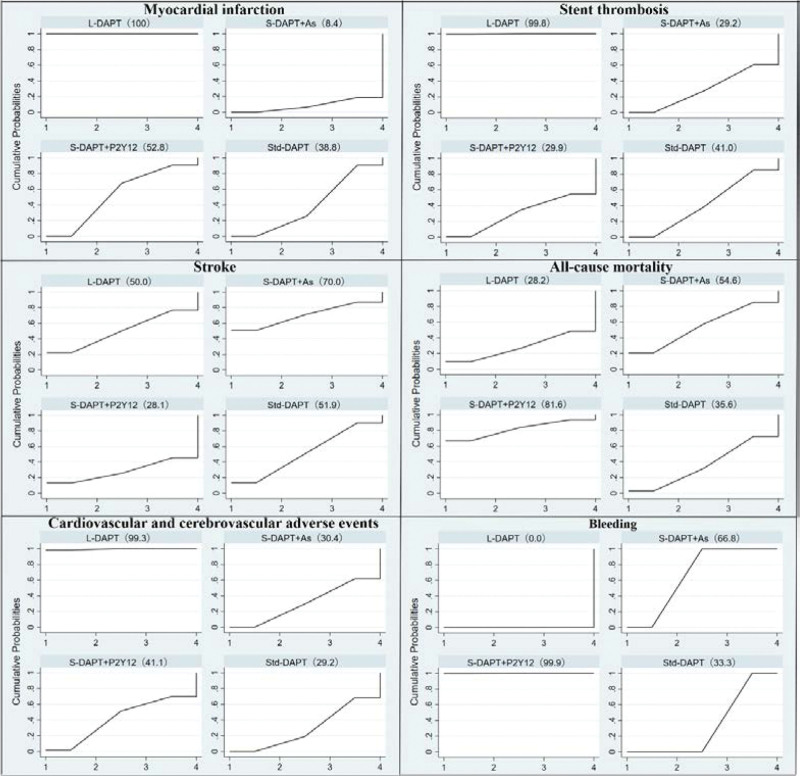
The result of SUCRA sequencing. SUCRA = surface under the cumulative ranking.

A total of 24 RCTs were included.^[18–21,23–28,30–43]^ Results of NMA showed that other 3 interventions were higher than L-DAPT (Std-DAPT [OR = 1.82, 95%CI: 1.49-2.21], S-DAPT + As [OR = 2.06, 95%CI: 1.57-2.70], S-DAPT + P2Y12 [OR = 1.71, 95%CI: 1.29-2.28]). There was no statistical significance among other interventions (Table 3). SUCRA sequencing results showed that: L-DAPT (100.0) > S-DAPT + P2Y12 (52.8) > Std-DAPT (38.8) > S-DAPT + As (8.4) (Fig. 3).

#### 3.4.2. Stent thrombosis.

A total of 25 RCTs were included.^[18,20–38,40–43]^ Results of NMA showed that other 3 interventions were higher than L-DAPT [Std-DAPT (OR = 2.18, 95%CI: 1.45-3.28), S-DAPT + As (OR = 2.32, 95%CI: 1.52-3.54), S-DAPT + P2Y12 (OR = 2.31, 95%CI: 1.22-4.36)]. There was no statistical significance among other interventions (Table 3). SUCRA sequencing results showed that: L-DAPT (99.8) > Std-DAPT (41.0) > S-DAPT + P2Y12 (29.9) > S-DAPT + As (29.2) (Fig. 3).

#### 3.4.3. Stroke.

A total of 20 RCTs were included.^[18,21–23,25–28,30–41]^ Results of NMA showed that there was no statistical significance among 4 interventions (Table 3). SUCRA sequencing results showed that: S-DAPT + As (70.0) > Std-DAPT (51.9) > L-DAPT (50.0) > S-DAPT + P2Y12 (28.1) (Fig. 3).

#### 3.4.4. All-cause mortality.

A total of 25 RCTs were included.^[18–28,30–43]^ Results of NMA showed that there was no statistical significance among 4 interventions (Table 3). SUCRA sequencing results showed that: S-DAPT + P2Y12 (81.6) > S-DAPT + As (54.6) > Std-DAPT (35.6) > L-DAPT (28.2) (Fig. 3).

#### 3.4.5. Cardiovascular and cerebrovascular adverse events.

A total of 27 RCTs were included.^[18–43]^ Results of NMA showed that other 3 interventions were higher than L-DAPT (Std-DAPT [OR = 1.28, 95%CI: 1.12-1.45], S-DAPT + As [OR = 1.27, 95%CI: 1.09-1.48], S-DAPT + P2Y12 [OR = 1.24, 95%CI: 1.01-1.52]). There was no statistical significance among other interventions (Table 3). SUCRA sequencing results showed that: L-DAPT (99.3) > S-DAPT + P2Y12 (41.1) > S-DAPT + As (30.4) > Std-DAPT (29.2) (Fig. 3).

#### 3.4.6. Bleeding.

A total of 27 RCTs were included.^[18–43]^ Results of NMA showed that other 3 interventions were lower than L-DAPT (Std-DAPT [OR = 0.67, 95%CI: 0.52-0.85], S-DAPT + As [OR = 0.51, 95%CI: 0.39-0.66], S-DAPT + P2Y12 [OR = 0.36, 95%CI: 0.26-0.49]). Two interventions were lower than L-DAPT (S-DAPT + As [OR = 0.77, 95%CI: 0.65-0.90], S-DAPT + P2Y12 [OR = 0.54, 95%CI: 0.44-0.66]). S-DAPT + As was higher than L-DAPT (OR = 1.42, 95%CI: 1.10-1.83) (Table 3). SUCRA sequencing results showed that: S-DAPT + P2Y12 (99.9) > S-DAPT + As (66.8) > Std-DAPT (33.3) > L-DAPT (0.0) (Fig. 3).

### 3.5. Risk assessment of bias

**Figure 4.**
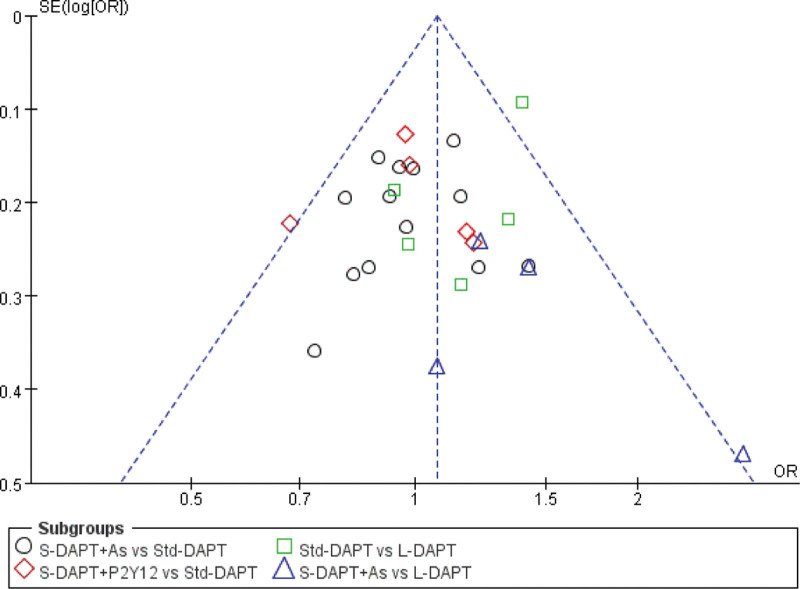
The funnel plot.

The funnel plot was drawn for publication bias test for the outcome index of cardiovascular and cerebrovascular adverse events. The results showed that the distribution of each study point was roughly symmetrical on both sides of the funnel plot, suggesting that there was little possibility of publication bias (The funnel plot in Fig. 4).

## 4. Discussion

As P2Y12 receptor inhibitors show extensive individual differences among different individuals, especially clopidogrel, about one-third of patients receiving clopidogrel will develop platelet resistance.^[44,45]^ At the same time, platelet resistance may be associated with higher cardiogenic death and ischemic events.^[46]^ Thus, aspirin is still the best choice for antiplatelet therapy. In recent years, despite the deepening understanding of the pathogenesis of coronary artery disease and the continuous optimization of PCI technology, there is still controversy about how to balance ischemia and bleeding after surgery and the choice of the best antiplatelet regimen. In clinical practice, the severity of bleeding is often underestimated relative to the risk of ischemia, and the bleeding risk of discharged patients after PCI may be greater than the ischemic risk of MI, and is bleeding directly related to death.^[47]^ Std-DAPT does reduce the risk of late thrombosis after PCI, but it also brings about safety of bleeding, so the duration of DAPT has been the focus of attention.^[48]^ Current European, American and Chinese guidelines^[7–9]^ all recommend that patients with high bleeding risk should receive S- DAPT + As due to the bleeding problems caused by Std-DAPT and L-DAPT.

Our study showed that the bleeding risk of S-DAPT + As was significantly lower than Std-DAPT and L-DAPT. There was no statistical significance in the efficacy of S-DAPT + As, Std-DAPT and S-DAPT + P2Y12. In the outcome of MI, stent thrombosis, and cardiovascular and cerebrovascular adverse events, the efficacy of S-DAPT + As was inferior to L- DAPT.

More than two decades ago, the addition of the P2Y12 receptor inhibitor clopidogrel to initial aspirin monotherapy treatment was shown to reduce the risk of ischemia in patients with non-ST-segment elevation ACS, but also to increase the risk of major bleeding.^[49]^ In recent years, the efficacy of aspirin has been greatly challenged. Armstrong demonstrated that in the presence of an effective P2Y12 receptor inhibitor, aspirin had little additional inhibitory effect on thromboxane A2-mediated platelet aggregation.^[50]^ Studies have shown that the effect of P2Y12 receptor inhibitor monotherapy to the coagulation system is similar to that of DAPT.^[51]^ The MATCH trial^[52]^ confirmed that in high-risk stroke patients, clopidogrel monotherapy may reduce ischemic risk as much as DAPT, with a lower risk of bleeding. Our study showed that S-DAPT + P2Y12 had the least bleeding risk. There was no statistical significance in the efficacy of S-DAPT + P2Y12, S-DAPT + As, Std-DAPT. In the outcome of MI, stent thrombosis, and cardiovascular and cerebrovascular adverse events, the efficacy of S-DAPT + P2Y12 was inferior to L-DAPT.

ACS patients with particularly complex coronary lesions, especially those with diabetes, have a high risk of ischemia after PCI, and 10% of ischemic events still occur after Std-DAPT therapy.^[53]^ The optimal duration of DAPT has been controversial since the advent of DES. Initially in patients following rapamycin DES implantation, a minimum duration of 3 months of DAPT was recommended. DAPT was recommended for a minimum duration of 6 months after implantation of paclitaxel DESs. Afterwards, regardless of the type of DES, an extension of the DAPT duration to 1 year or more was recommended to reduce the risk of thrombosis as reported in some observational studies.^[54,55]^ Therefore, it is necessary for meta-analysis to comprehensively evaluate L-DAPT treatment regimen. Our study showed that L-DAPT had the greatest bleeding risk. In the outcome of MI, stent thrombosis, and cardiovascular and cerebrovascular adverse events, L-DAPT had the best efficacy. In the outcome of stroke and all-cause mortality, there was no statistical significance among 4 interventions.

Some limitations of this study exist. First, there are many types of P2Y12 receptor inhibitors, and their efficacy varies greatly. Due to the limitations of the included studies, subgroup analysis cannot be performed, which may affect the reliability of the conclusion. Second, the subtypes, complications and interventional procedure of coronary heart disease in the included patients were different, which may affect the conclusions. Lastly, most of the included studies were not blinded, and there may be biases in implementation and measurement.

Due to the influence of indirection of evidence and sample size, it is hoped that there will be more RCTs in the future to verify the relationship between its efficacy and safety.

## 5. Conclusions

The available evidence suggests that S-DAPT + P2Y12 has the lowest bleeding risk, while L-DAPT has the highest bleeding risk. In the outcome of MI, stent thrombosis, and cardiovascular and cerebrovascular adverse events, L-DAPT has the best efficacy. In the outcome of stroke and all-cause mortality, the 4 interventions were equally effective.

## Author contributions

**Conceptualization:** Lin Luo, Shenglin Wang, Kai Tang.

**Data curation:** Lin Luo, Shenglin Wang, Kai Tang, Liqiong Xu.

**Formal analysis:** Lin Luo, Shenglin Wang, Kai Tang, Jianli Wu, Liqiong Xu.

**Funding acquisition:** Shenglin Wang, Kai Tang, Jianli Wu, Liqiong Xu.

**Investigation:** Shenglin Wang, Kai Tang, Tao Feng, Dejin Li.

**Methodology:** Shenglin Wang, Kai Tang, Tao Feng, Jiuju Ran.

**Project administration:** Shenglin Wang, Kai Tang, Xu Yang, Tao Feng, Li Zhang.

**Resources:** Shenglin Wang, Kai Tang, Dan Wang, Dan Zhao.

**Software:** Shenglin Wang, Kai Tang, Xu Yang, Dan Zhao.

**Supervision:** Shenglin Wang, Kai Tang, Xu Yang, Debo Li.

**Validation:** Lin Luo, Shenglin Wang, Kai Tang, Debo Li.

**Visualization:** Lin Luo, Shenglin Wang, Kai Tang.

**Writing – original draft:** Lin Luo.

**Writing – review & editing:** Lin Luo.

## Supplementary Material


