# Supplementary material for: Efficacy and safety of dual antiplatelet therapy after percutaneous coronary drug-eluting stenting: A network meta-analysis
Source: Medicine (Baltimore). 2022 Oct 21;101(42):e31158. doi: 10.1097/MD.0000000000031158 (PMC9592305; doi:10.1097/MD.0000000000031158)
Supplement: Supplementary file 1 [file medi-101-e31158-s001.pdf]

Search strategy by PubMed

#1 Percutaneous Coronary Intervention [Mesh Terms]

#2 PCI [Title/Abstract] OR Percutaneous Coronary Interventions [Title/Abstract] OR Coronary Intervention，Percutaneous [Title/Abstract]

#3 #1 OR #2

#4 Platelet Aggregation Inhibitors [Mesh Terms] OR Dual Anti-Platelet Therapy [Mesh Terms]

#5 DAPT [Title/Abstract] OR Dual Antiplatelet Therapy [Title/Abstract] OR SAPT [Title/Abstract] OR Single Antiplatelet Therapy [Title/Abstract] OR Antiplatelet [Title/Abstract] OR Aspirin [Title/Abstract] OR Clopidogrel [Title/Abstract] OR Ticagrelor [Title/Abstract] OR Prasugrel [Title/Abstract] OR Ticlopidine [Title/Abstract] OR P2Y12 Inhibitor [Title/Abstract]

#6 #4 OR #5

#7 Randomized Controlled Trial [Mesh Terms]

#8 Randomized Controlled Trial [Publication Type] OR Randomized Controlled Trial [All Fields] OR RCT [All Fields]

#9 #7 OR #8

#10 #3 AND #6 AND #9
